# Correction: Implementing WHO guidance on conducting and analysing vaccination coverage cluster surveys: Two examples from Nigeria

**DOI:** 10.1371/journal.pone.0253670

**Published:** 2021-06-17

**Authors:** John Ndegwa Wagai, Dale A. Rhoda, Mary L. Prier, Mary Kay Trimner, Caitlin B. Clary, Joseph Oteri, Bassey Okposen, Adeyemi Adeniran, M. Carolina Danovaro-Holliday, Felicity T. Cutts

There are multiple spelling errors in the author list. Please see the correct author list and citation here:

John Ndegwa Wagai, Dale A. Rhoda, Mary L. Prier, Mary Kay Trimner, Caitlin B. Clary, Joseph Oteri, Bassey Okposen, Adeyemi Adeniran, M. Carolina Danovaro-Holliday, Felicity T. Cutts

Wagai JN, Rhoda DA, Prier ML, Trimner MK, Clary CB, et al. (2021) Implementing WHO guidance on conducting and analysing vaccination coverage cluster surveys: Two examples from Nigeria. PLoS ONE 16(2): e0247415. https://doi.org/10.1371/journal.pone.0247415

The ORCID iDs are missing for the ninth and tenth author. Please see the authors’ respective ORCID iDs here:

- Author M. Carolina Danovaro-Holliday’s ORCID iD is: 0000-0001-7324-9198 (https://orcid.org/0000-0001-7324-9198).
- Author Felicity T. Cutts’ ORCID iD is: 0000-0002-8834-1146 (https://orcid.org/0000-0002-8834-1146).
