# Supplementary figures and images for: Implementing WHO guidance on conducting and analysing vaccination coverage cluster surveys: Two examples from Nigeria
Source: PLoS One. 2021 Feb 26;16(2):e0247415. doi: 10.1371/journal.pone.0247415 (PMC7909665; doi:10.1371/journal.pone.0247415)

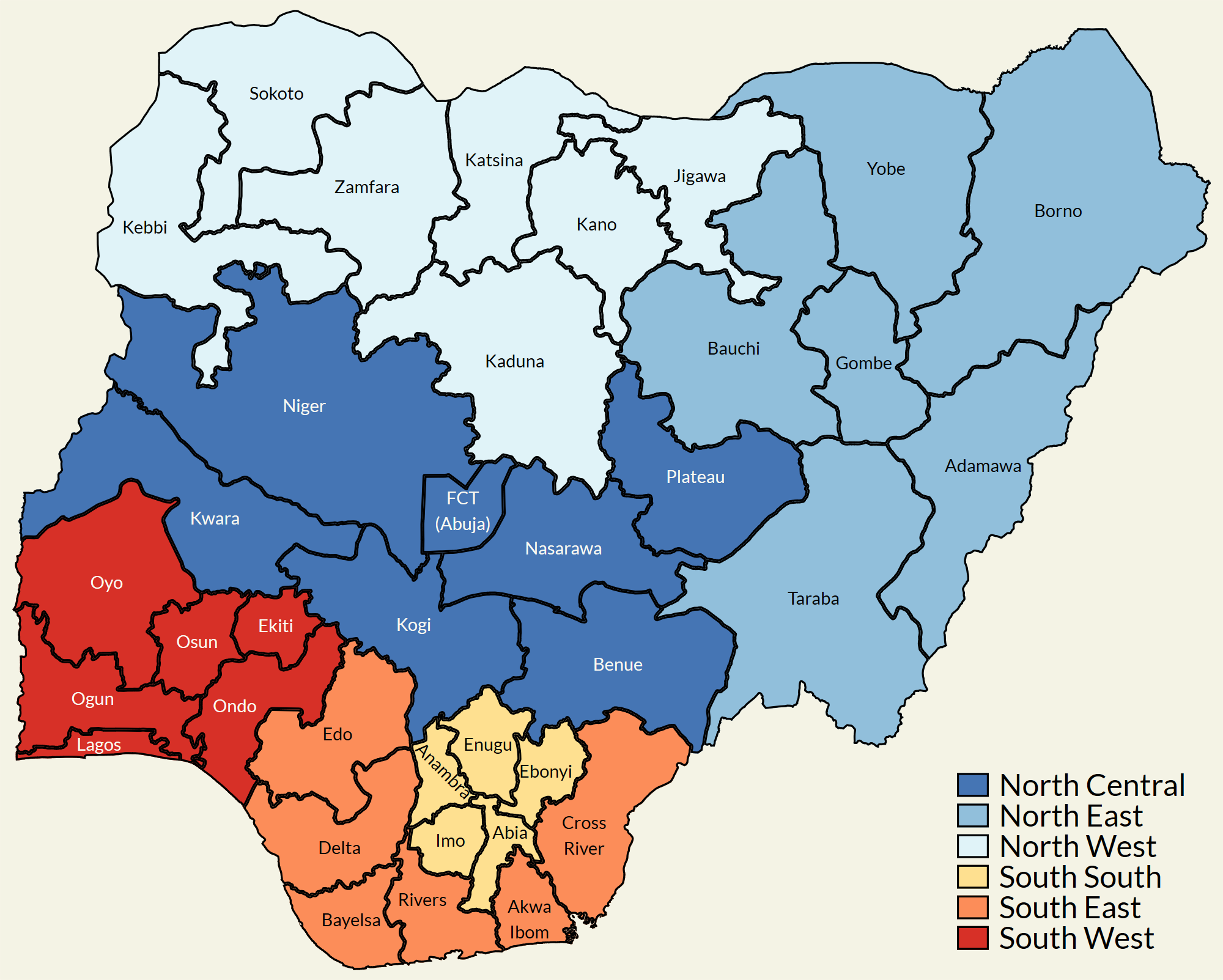

Supplement: S1 Fig — (TIF) [file pone.0247415.s001.tif]

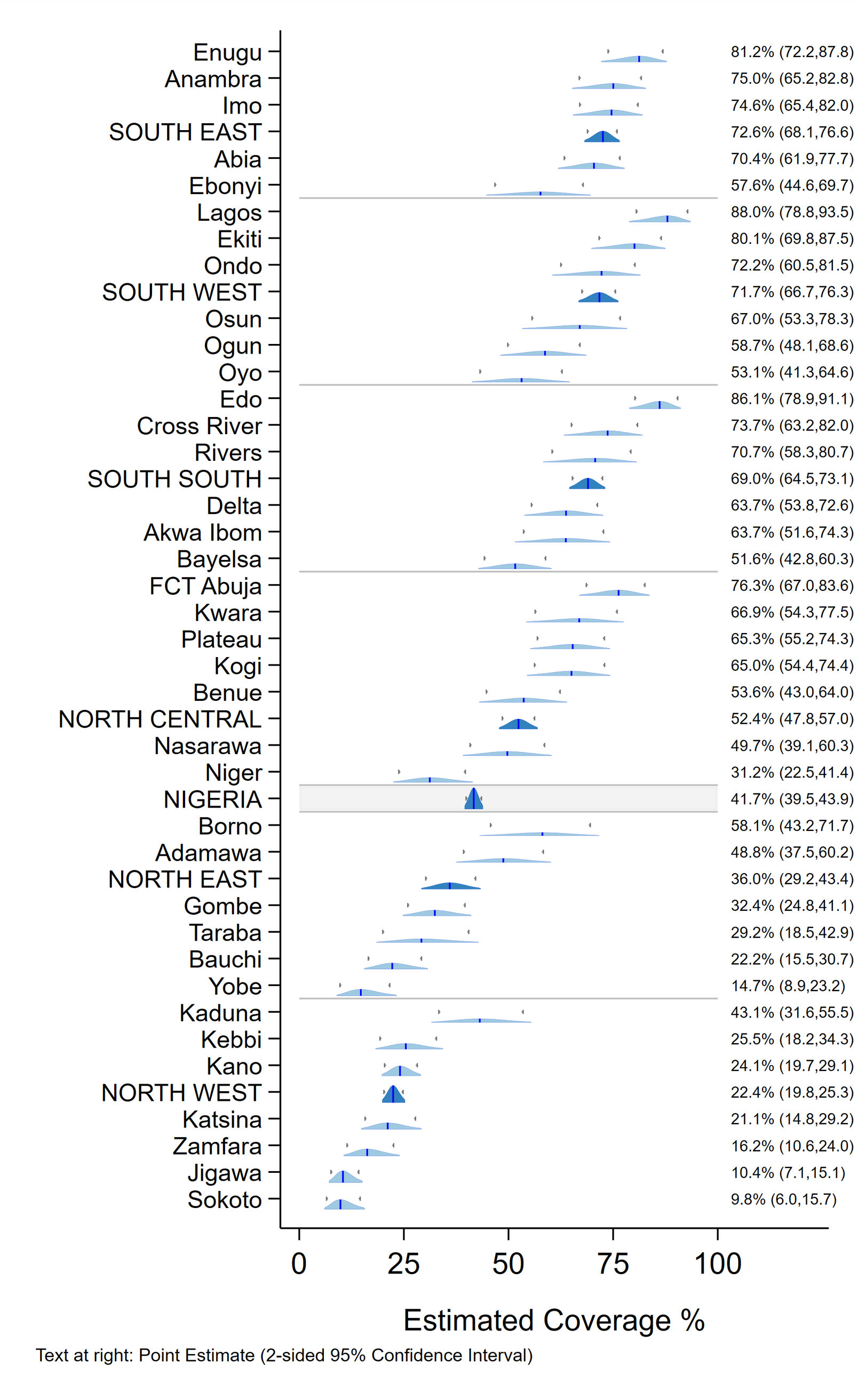

Supplement: S2 Fig — (TIF) [file pone.0247415.s002.tif]

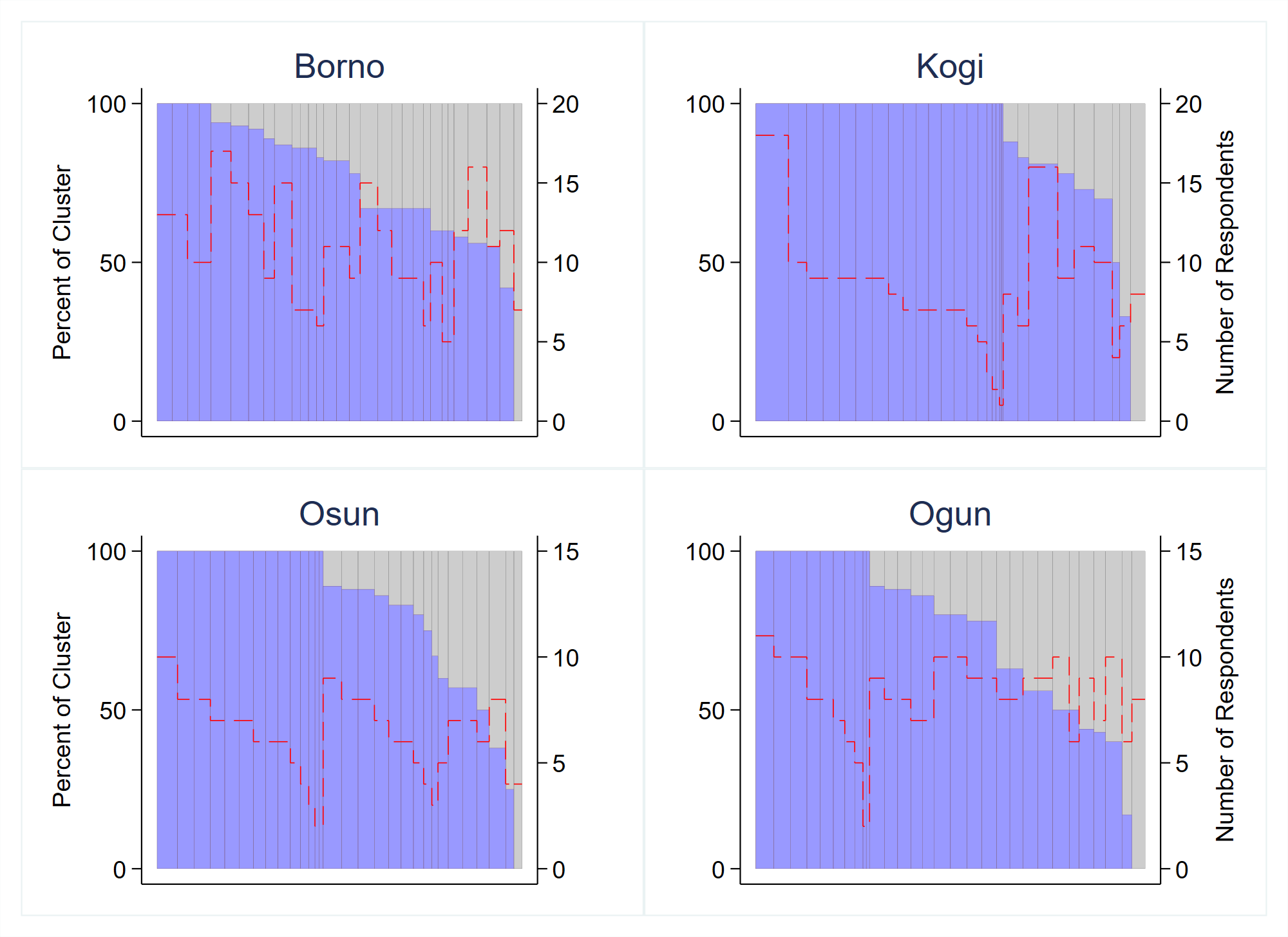

Supplement: S3 Fig — (TIF) [file pone.0247415.s003.tif]

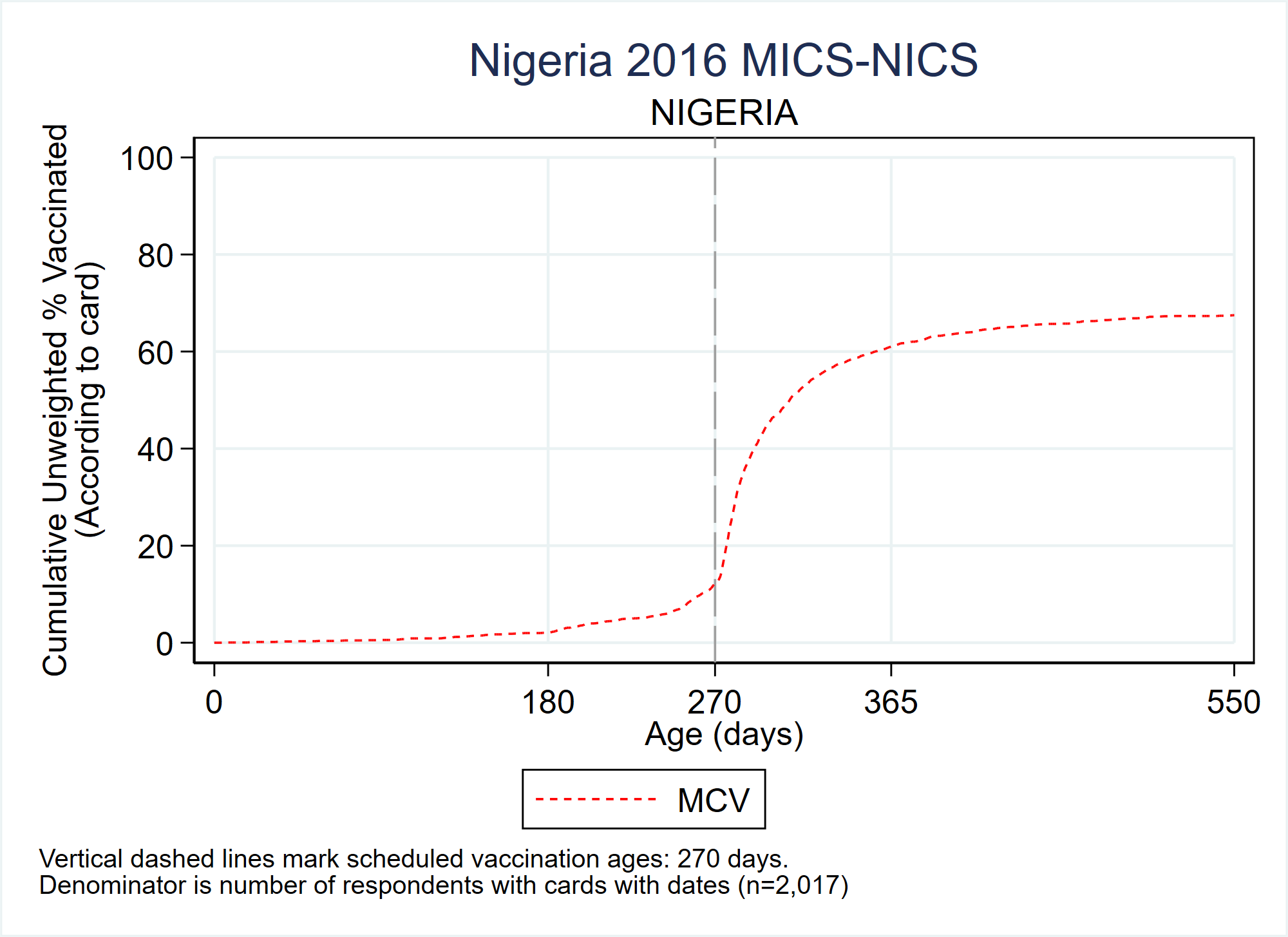

Supplement: S4 Fig — (TIF) [file pone.0247415.s004.tif]

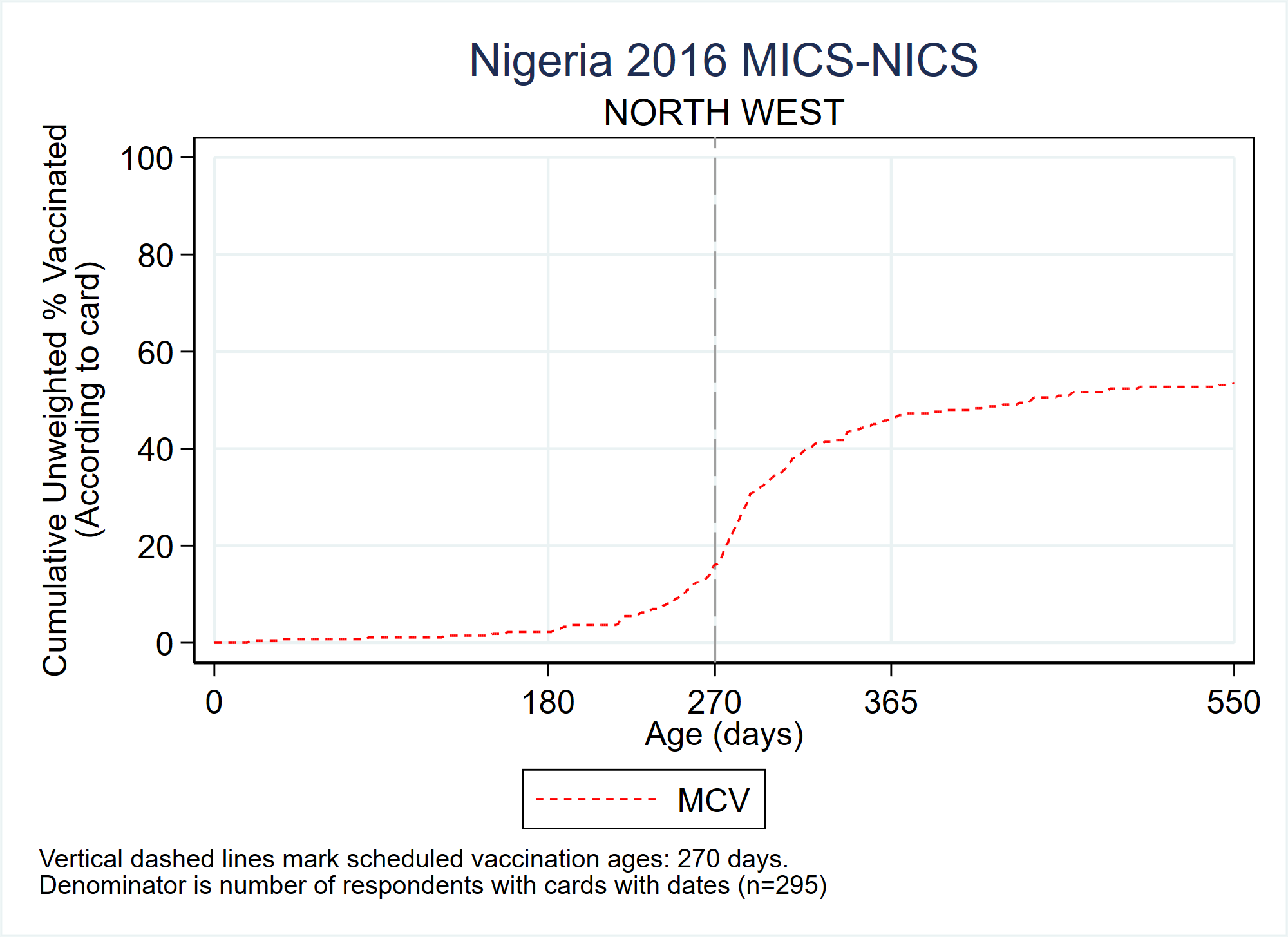

Supplement: S5 Fig — (TIF) [file pone.0247415.s005.tif]

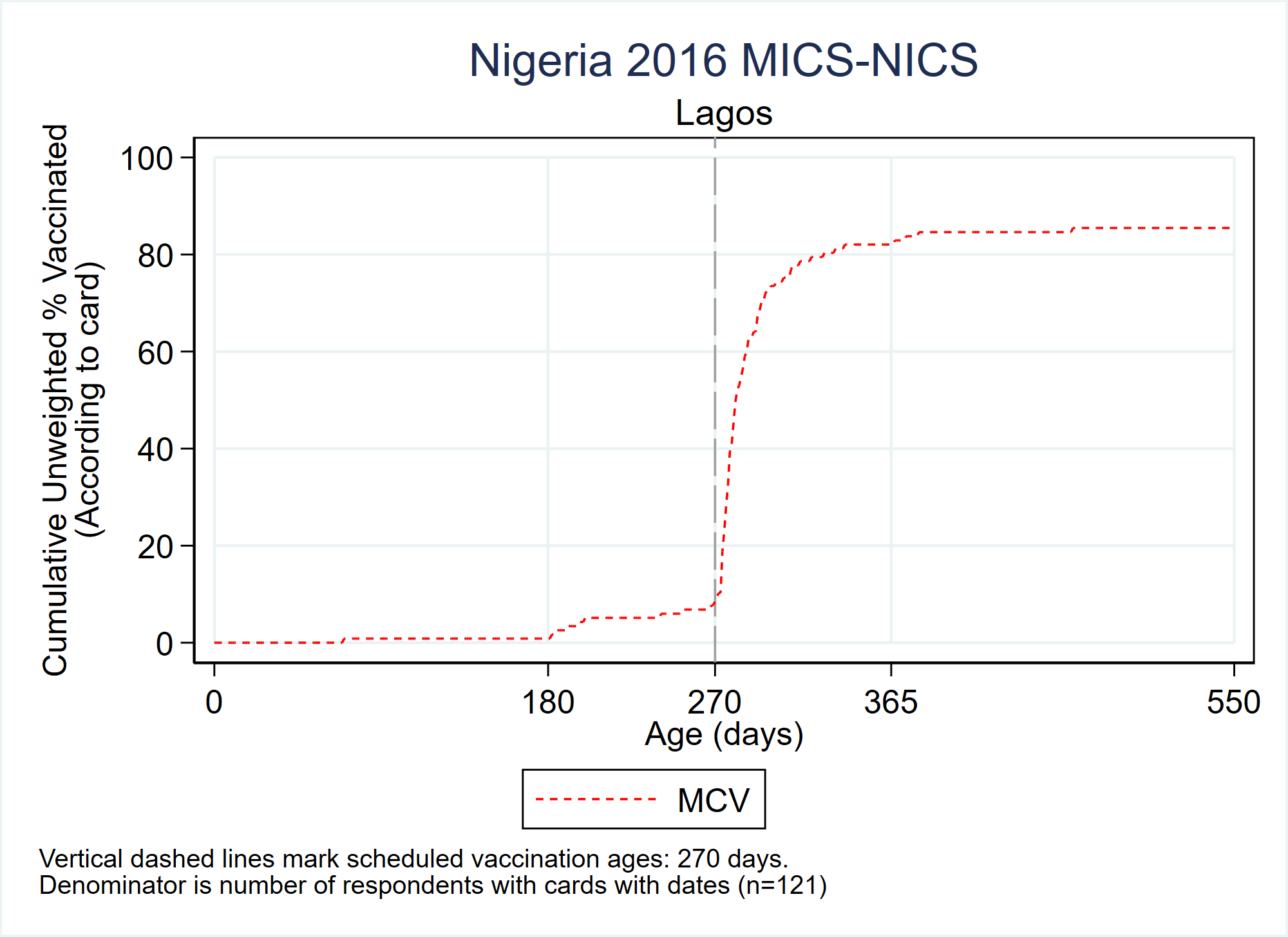

Supplement: S6 Fig — (TIF) [file pone.0247415.s006.tif]

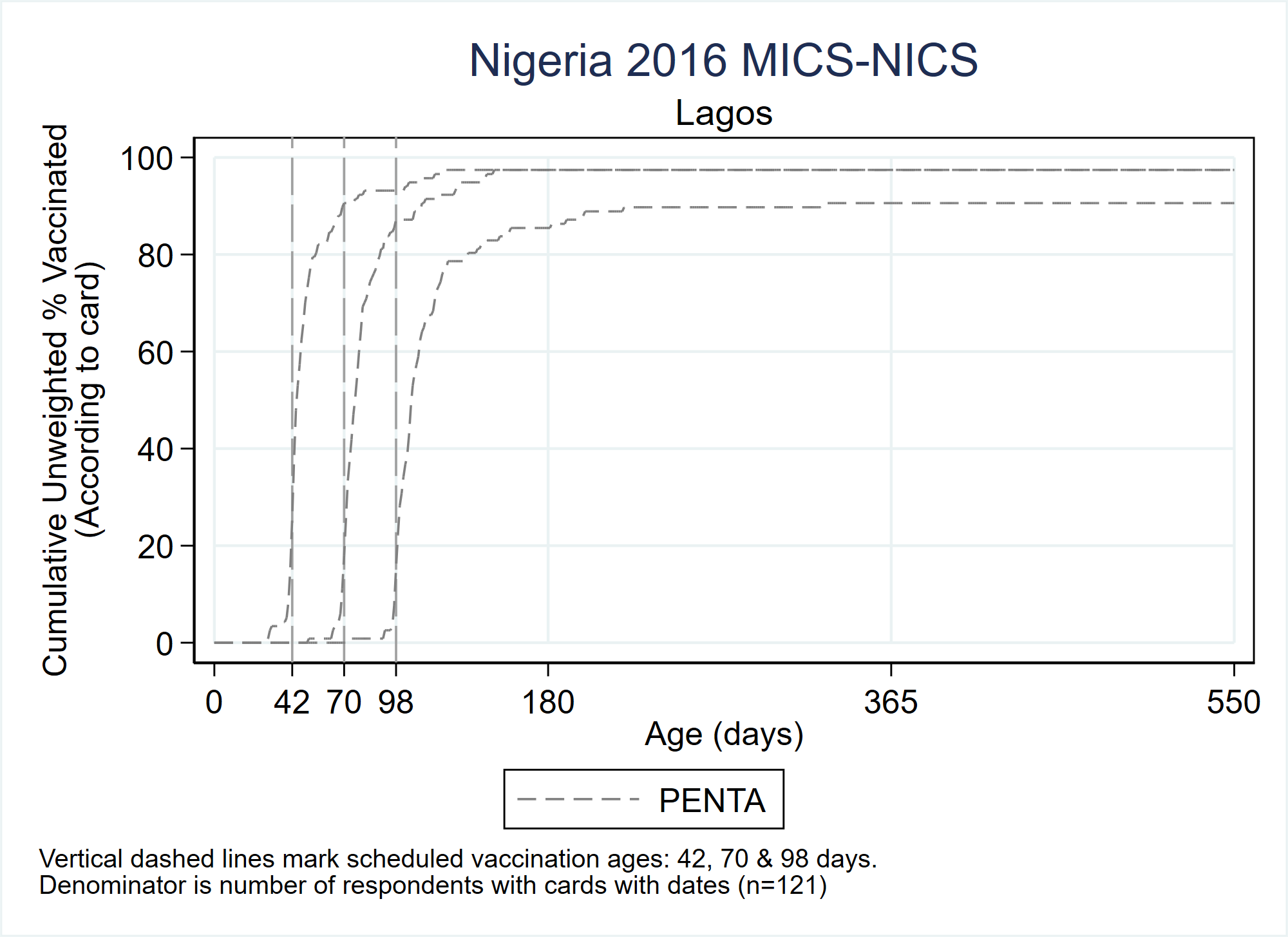

Supplement: S7 Fig — (TIF) [file pone.0247415.s007.tif]

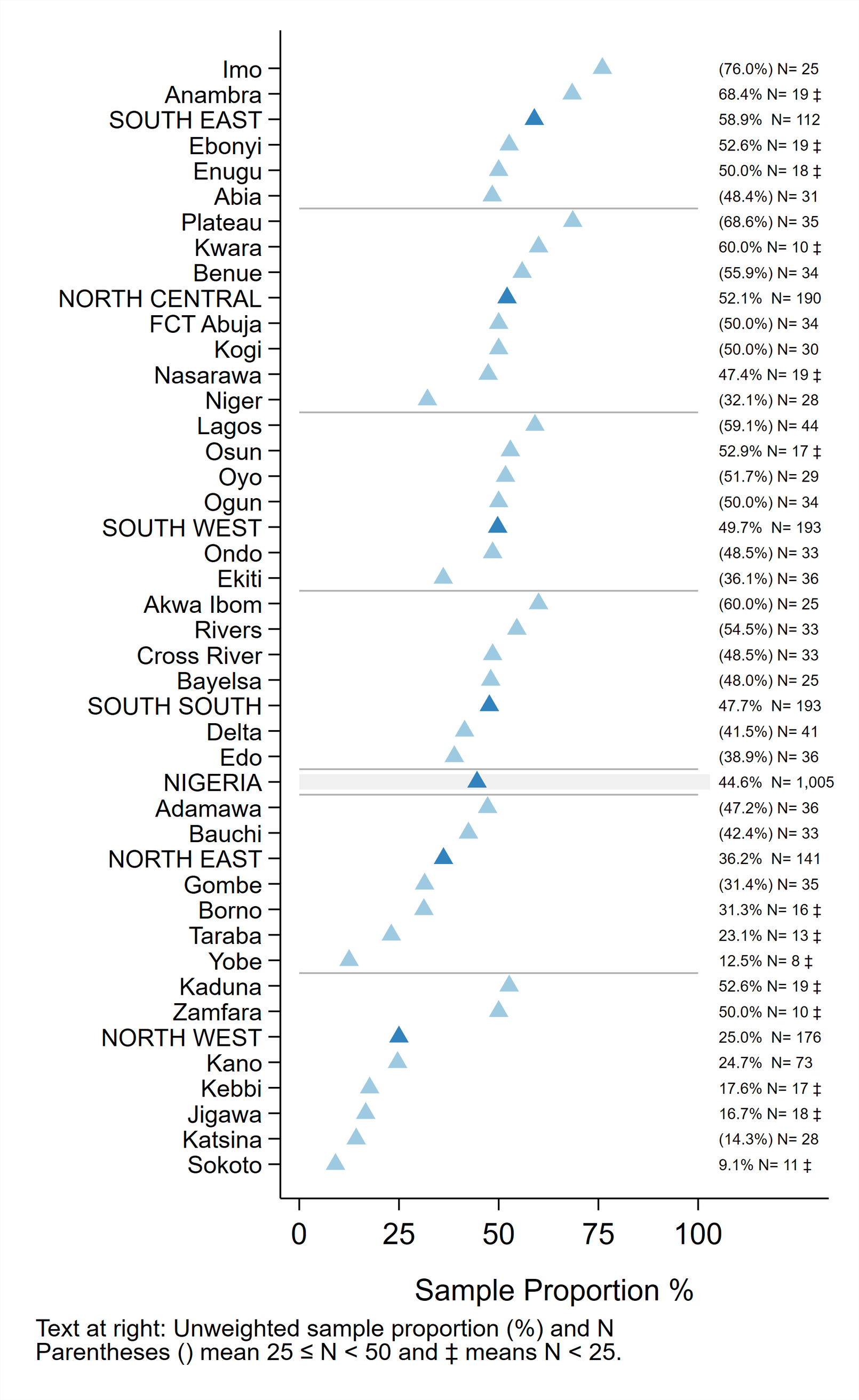

Supplement: S8 Fig — (TIF) [file pone.0247415.s008.tif]

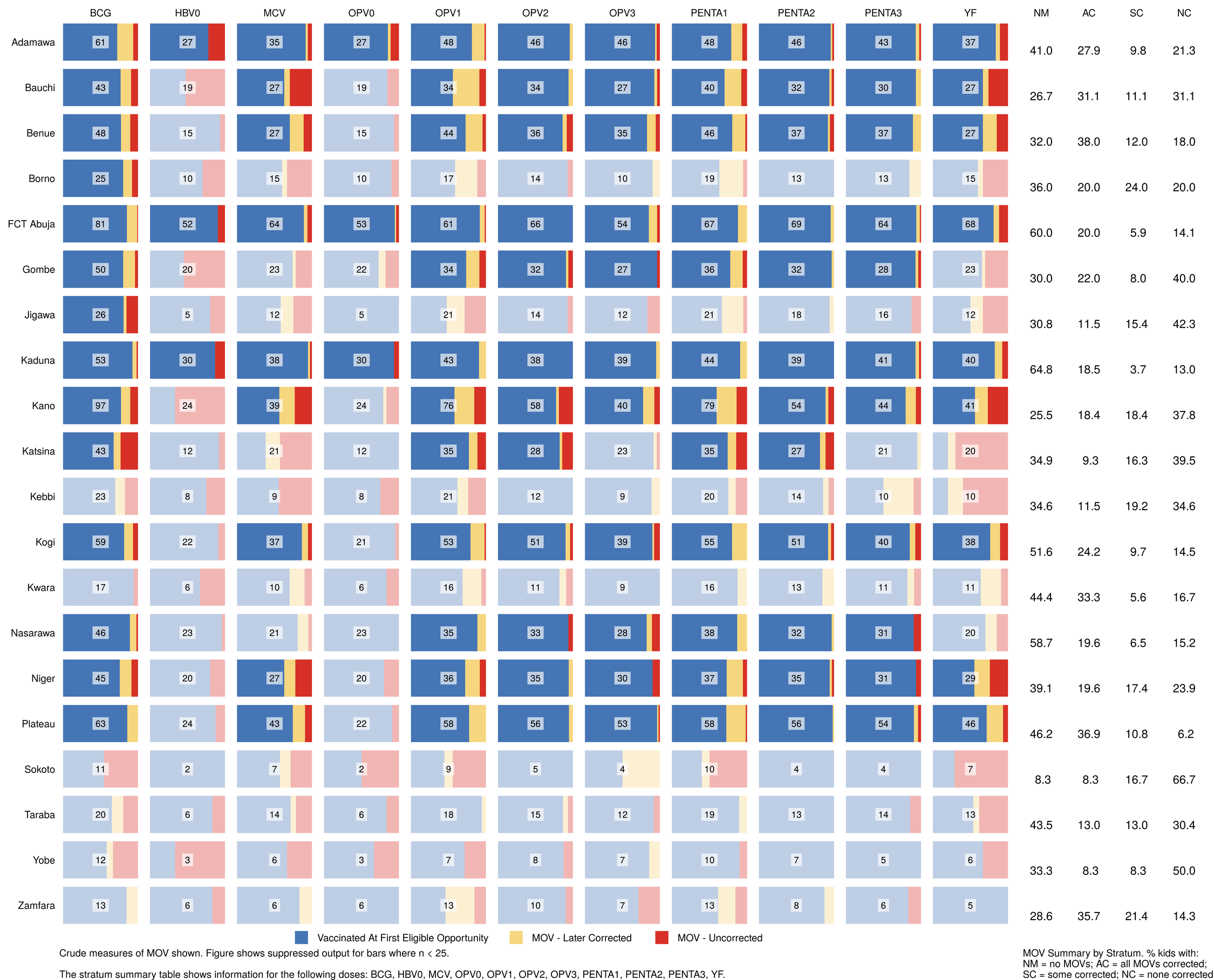

Supplement: S9 Fig — (TIF) [file pone.0247415.s009.tif]

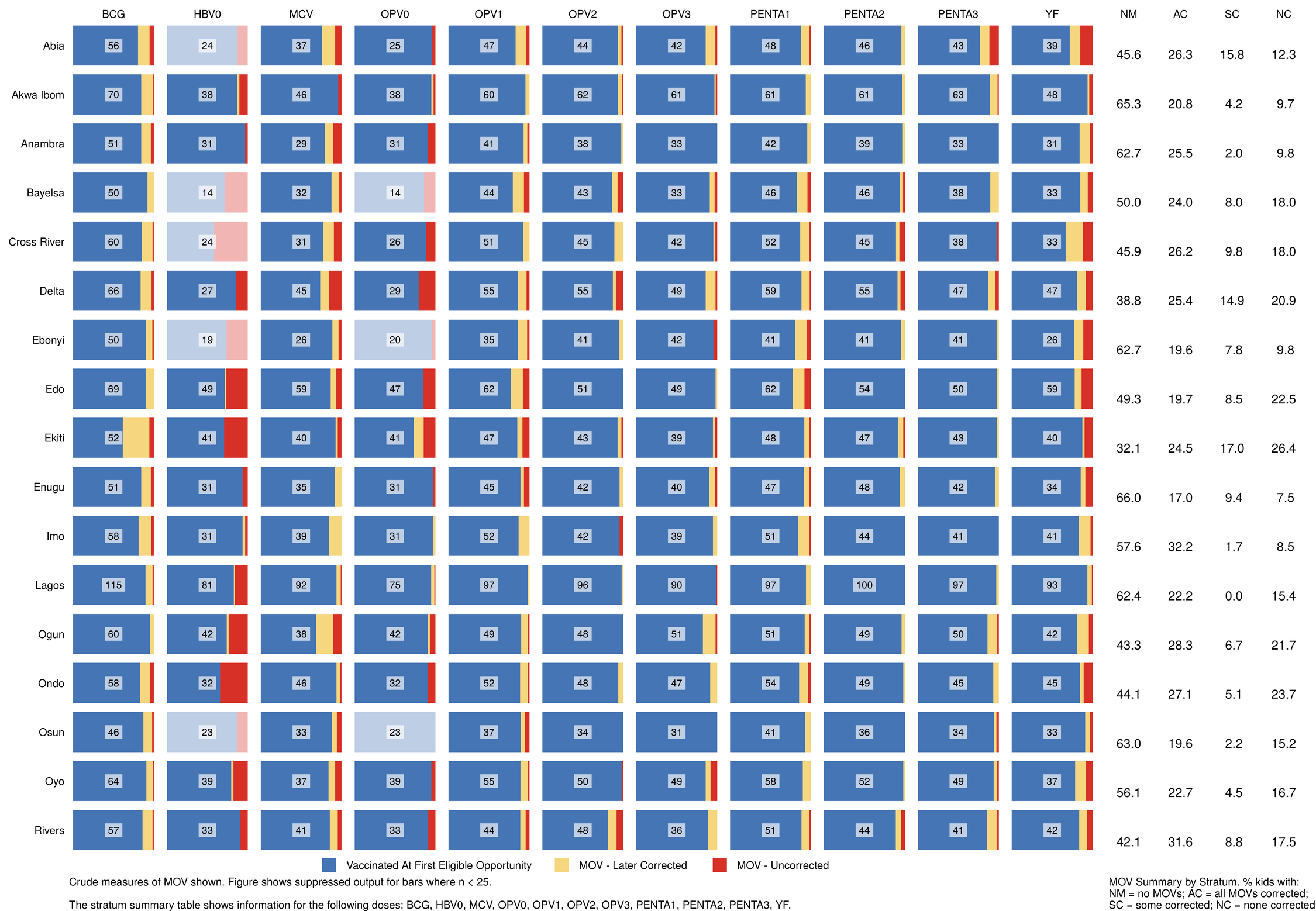

Supplement: S10 Fig — (TIF) [file pone.0247415.s010.tif]
