# Supplementary material for: Implementing WHO guidance on conducting and analysing vaccination coverage cluster surveys: Two examples from Nigeria
Source: PLoS One. 2021 Feb 26;16(2):e0247415. doi: 10.1371/journal.pone.0247415 (PMC7909665; doi:10.1371/journal.pone.0247415)
Supplement: S1 Table — (DOCX) [file pone.0247415.s011.docx]

**S1 Table: Selected definitions used in the Vaccination Coverage Quality Indicators (VCQI) tool for indicators calculated using weighted analyses**

| **Indicator** | **Definition** | **Numerator** | **Denominator** |
| --- | --- | --- | --- |
| Home-based record (HBR) ever ownership | Percentage of children age 12-23 months whose mother or caretaker says have ever received a HBR, even if the record is not seen on the day of the interview (e.g. because it is locked away and the person with the key is absent, or it was lost) | Sum of weights of children said to have had a vaccination record even if not seen on day of interview | Sum of weights of all children in the sample in the defined target population (12-23 months) |
| HBR current ownership | Percentage of children age 12-23 months whose home-based record is seen and contains usable information | Sum of weights of children whose vaccination record is seen and has at least one date of vaccination on it | Sum of weights of all children aged 12-23 months in the sample |
| Crude Coverage of {vaccine-dose} * | Percentage of children age 12-23 months who received {vaccine-dose} at any age by time of survey (sub-categories: according to documentation (HBR) and according to maternal recall) | Sum of weights of all children identified in the denominator who received the specified vaccine dose(s) (by sub-categories: by documented evidence or by recall) by any date prior to the survey | Sum of weights of all children in the sample in the defined target population (12-23 months) |
| Crude Coverage of fully vaccinated** children | Percentage of children age 12-23 months who received all vaccine-doses recommended in the national schedule by time of survey (sub-categories: according to documentation (HBR) and according to maternal recall) | Sum of weights of all children identified in the denominator who received all vaccine-doses included in the fully vaccinated definition | Sum of weights of all children in the sample in the defined target population (12-23 months) |
| Valid Coverage of {vaccine-dose} * based on documented evidence | Percentage of children age 12-23 months who received {vaccine-dose} respecting the minimum age for each dose and the minimum interval between doses as recommended in the national schedule by time of survey. Because documented dates of each vaccine-dose are needed in order to determine if the schedule was followed appropriately, this can only be calculated among children with documented evidence. | Sum of the sample weights for children 12-23 months who are vaccinated where the doses followed the earliest recommended age and minimum interval between doses | Sum of the sample weights for children 12-23 months with documented evidence of their vaccination status  (unless otherwise specified – can have a second related indicator that includes all children in denominator) |
| Never vaccinated (with any of the basic antigens) coverage | Percentage of children aged 12-23 months who received none of the basic vaccines included in the country schedule | Sum of the sample weights for those of children who received none of recommended vaccine doses for the basic six antigens, by the time of the survey, based on the combination of recall and documented evidence | Sum of weights of all children aged 12-23 months in the sample |
| Supplementary Immunization Activity (SIA) coverage {in this case with measles-containing vaccine (MCV)} | Percentage of children aged 9 months to 59 months {or age group targeted in the SIA} who received a dose of MCV during the SIA | Sum of weights of children said to have received MCV during the SIA | Sum of weights of all children aged 9-59 months in the sample |
| Percentage of children not vaccinated against measles before SIA (“zero-dose” children) | Percentage of children aged 9 months to 59 months who had not received a dose of MCV before the SIA | Sum of weights of children with no history or record of receiving MCV before the SIA | Sum of weights of all children aged 9-59 months in the sample |
| Percentage of children already vaccinated against measles before SIA | Percentage of children aged 9 months to 59 months who had received a dose of MCV before the SIA | Sum of weights of children with a history or record of having received MCV before the SIA | Sum of weights of all children aged 9-59 months in the sample |
| SIA coverage among measles zero-dose children | Percentage of children aged 9-59 months at the time of the SIA with NO history of receipt of MCV before the SIA who received a dose of MCV during the SIA (by card, finger-mark or parental recall) | Sum of weights of children with no history or record of receiving MCV before the SIA who received a dose of MCV during the SIA | Sum of weights of children with no history or record of receiving MCV before the SIA |
| SIA coverage among children vaccinated  previously | Percentage of children aged 9-59 months at the time of the SIA with a history of receipt of MCV before the SIA who received a dose of MCV during the SIA (by card, finger-mark or parental recall) | Sum of weights of children with a history or record of having received MCV before the SIA who received a dose of MCV during the SIA | Sum of weights of children with a history or record of having received MCV before the SIA |

* {vaccine-dose} is specified in each row, e.g. BCG, pentavalent 1, pentavalent 2, etc.

** May have 2 definitions for fully vaccinated, one focusing only on the original 6 vaccines and another including all vaccines in the schedule – see results table.
