# Supplementary material for: Implementing WHO guidance on conducting and analysing vaccination coverage cluster surveys: Two examples from Nigeria
Source: PLoS One. 2021 Feb 26;16(2):e0247415. doi: 10.1371/journal.pone.0247415 (PMC7909665; doi:10.1371/journal.pone.0247415)
Supplement: S2 Table — (DOCX) [file pone.0247415.s012.docx]

**S2 Table: Selected definitions used in VCQI for indicators calculated using unweighted analyses which represent a subset of the total target population**

| Indicator | Definition | Numerator | Denominator |
| --- | --- | --- | --- |
| Percentage of {vaccine- doses} that were invalid | The percentage of children who had received the relevant {vaccine-dose} while they were either at too young or with too short an interval since the previous dose in the series.  E.g. DTP1<42 days; DTP2<70 days or <28 days after DTP1; DTP3<98 days or <28 days since DTP2; MCV1<273 days. | Number of respondents whose {vaccine-dose} was invalid | e.g. Number of respondents who had date of birth data within specified ranges and received {vaccine-dose} with a date |
| Dropout between vaccine-doses  (e.g. Penta1 to Penta3; Penta1 to MCV etc. | The estimated percentage of children 12-23 months who received the specified vaccine-dose given earliest in the schedule but failed to receive the specified subsequent vaccine-dose in the schedule. For example, Penta1 to Penta3 dropout is shown here: dropout = (Penta1-Penta3)/Penta1  This indicator can be sub-categorized according to source of evidence (HBR/recall/either) | Number of respondents who received the first dose but did not receive the later dose  (e.g. received Penta1 but did not receive Penta3) | Number of respondents who received the first dose (e.g. Penta1) |
| Percent of children with missed opportunity for simultaneous vaccination (MOV) | Percent of children who on at least on occasion, did not receive all the vaccines for which they were eligible*. E.g. those with different dates for Penta1 and OPV1.  Can sub-categorise according to whether the MOV was later corrected or not (i.e., was the missed vaccine-dose received later at a valid age?)  Calculate for each vaccine and dose  Calculate over all vaccines and doses | Number of children who experienced 1+ missed opportunities to be vaccinated for the dose in question | Number of children with date of birth data within specified ranges and date of vaccination data indicating that they had 1+ visits for vaccination on days when they were eligible to receive the dose in question |
| Percent of *visits* with missed opportunity for simultaneous vaccination (MOV) | Percent of visits (i.e. considering all visits by all children with documentation) where at least one vaccine-dose was not administered despite the child being eligible*  Calculate for each vaccine and dose  Calculate over all vaccines and doses | Number of vaccination visit dates where a respondent did not receive all vaccinations for which they were eligible | Number of vaccination visit dates where a respondent was eligible to receive 1+ vaccinations |

*Eligibility is based on age of child and, where relevant, interval since the previous dose in the sequence. Information on any potential contraindications on the visit is not available hence all children are assumed to have no contraindications.
