# Supplementary material for: Implementing WHO guidance on conducting and analysing vaccination coverage cluster surveys: Two examples from Nigeria
Source: PLoS One. 2021 Feb 26;16(2):e0247415. doi: 10.1371/journal.pone.0247415 (PMC7909665; doi:10.1371/journal.pone.0247415)
Supplement: S3 Table — (DOCX) [file pone.0247415.s013.docx]

**S3 Table: Dropout rates between different vaccine-dose combinations in the vaccination series, by source of information, Nigeria MICS/NICS 2016-17.**

|  |  | Source of Information | | | | | |
| --- | --- | --- | --- | --- | --- | --- | --- |
|  |  | Children with HBR having received at least Penta1 | | Children without HBR having received at least Penta1 by recall | | All children with any evidence of having received Penta1 | |
|  |  | Penta1 to Penta3 | Penta1 to measles | Penta1 to Penta3 | Penta1 to measles | Penta1 to Penta3 | Penta1 to measles |
|  |  | % (weighted N) | % (weighted N) | % (weighted N) | % (weighted N) | % (weighted N) | % (weighted N) |
|  | National | 16.9 (1737) | 26.9 (1737) | 51.6 (1326) | 14.8 (1326) | 31.7 (3055) | 21.1 (3055) |
|  | Urban | 12.2 (797) | 18.7 (797) | 48.3 (571) | 9.3 (571) | 26.9 (1366) | 14.0 (1366) |
|  | Rural | 20.9 (940) | 33.9 (940) | 54.2 (755) | 18.9 (755) | 35.6 (1689) | 26.9 (1689) |
| Zone | North West | 28.8 (356) | 36.7 (356) | 69.5 (271) | 17.4 (271) | 46.1 (625) | 28.2 (625) |
|  | North East | 24.2 (288) | 36.7 (288) | 51.9 (343) | 17.4 (343) | 39.6 (629) | 26.3 (629) |
|  | North Central | 16.8 (276) | 26.9 (276) | 52.0 (253) | 10.4 (253) | 33.4 (528) | 18.2 (528) |
|  | South West | 7.2 (394) | 16.7 (394) | 45.6 (177) | 14.3 (177) | 18.7 (570) | 15.1 (570) |
|  | South East | 11.0 (164) | 23.1 (164) | 39.0 (138) | 15.6 (138) | 23.3 (301) | 19.1 (301) |
|  | South South | 11.1 (259) | 20.8 (259) | 36.4 (144) | 11.4 (144) | 19.3 (401) | 16.1 (401) |
| Caregiver's education | Non-formal | 36.1 (114) | 56.6 (114) | 66.0 (150) | 11.4 (150) | 53.7 (260) | 31.3 (260) |
|  | Primary | 16.2 (283) | 33.0 (283) | 49.4 (211) | 15.8 (211) | 30.1 (493) | 24.5 (493) |
|  | Secondary / Secondary-technical | 13.3 (803) | 22.6 (803) | 46.2 (520) | 13.8 (520) | 25.5 (1322) | 18.3 (1322) |
|  | Higher | 3.3 (260) | 7.1 (260) | 42.8 (193) | 4.8 (193) | 20.1 (452) | 5.7 (452) |
|  | Missing | 33.2 (276) | 39.8 (276) | 63.0 (252) | 25.6 (252) | 47.8 (527) | 33.4 (527) |
| Wealth index | Poorest | 31.6 (157) | 47.4 (157) | 67.5 (129) | 24.5 (129) | 47.7 (285) | 36.8 (285) |
|  | Second | 24.4 (243) | 39.0 (243) | 59.0 (231) | 22.0 (231) | 41.6 (470) | 30.7 (470) |
|  | Middle | 23.2 (348) | 32.7 (348) | 50.4 (244) | 11.6 (244) | 33.6 (591) | 23.3 (591) |
|  | Fourth | 14.1 (423) | 26.2 (423) | 44.2 (392) | 11.9 (392) | 28.4 (813) | 18.8 (813) |
|  | Richest | 7.8 (565) | 13.0 (565) | 50.1 (331) | 11.8 (331) | 23.1 (896) | 11.8 (896) |
|  | Median (range) by state* | 14.7 (3.0,45.5) | 26.9 (8.8,59.9) | 53.1 (19.6,94.4) | 11.8 (0,55.9) | 31.8 (13.4,69.0) | 20.6 (7.2,57.1) |

*The median, min. & max. percentages of the 37 states are provided, not the mean or weighted N.
