# Supplementary material for: Implementing WHO guidance on conducting and analysing vaccination coverage cluster surveys: Two examples from Nigeria
Source: PLoS One. 2021 Feb 26;16(2):e0247415. doi: 10.1371/journal.pone.0247415 (PMC7909665; doi:10.1371/journal.pone.0247415)
Supplement: S4 Table — (DOCX) [file pone.0247415.s014.docx]

**Supplementary Table 4: Data cleaning details for Nigeria MICS/NICS 2016-17 and PMCCS 2018**

| Issue | Resolution |
| --- | --- |
| Eligible population and indicator definitions | Vaccination coverage for the MICS/NICS focused on children aged 12-23m. The denominator for crude and valid coverage and fully-immunised and proportion showing cards is all eligible children in the survey. The denominator for indicators concerning timeliness and missed opportunities for vaccination (MOVs) is all eligible children whose date of birth was known, within specified ranges and showed a card with vaccination dates on it. The eligible population for the PMCCS is all children aged 9-59m and the denominator for coverage indicators is all eligible children in the PMCCS. |
| Steps to differentiate RI from SIA doses | The MICS/NICS asked not only about doses delivered through routine immunization but also, for children with a HBR that does not show a date of MCV, whether additional vaccines had been received in a campaign. For children without a HBR, mothers are asked if the child ever received each vaccine but without specifying if it was in RI or a campaign. For analysis purposes, if the HBR is blank for measles but the caretaker says the child participated in an SIA, the child could be given coverage credit based on the caretaker’s recall. This requires careful coding of the input data and tracking of provenance if the analysis plan calls for tables that document the sources of vaccination. |
| How valid doses are defined | A dose is *valid* if the child has reached the minimum age to receive the antigen and, for multi-dose antigens, if the appropriate interval has passed since the most recent dose. To assess age and interval duration, the software requires dates of birth and vaccination, so children who do not furnish HBRs and for whom FBRs are not found must be excluded from calculations about valid doses. There is a tradition in WHO EPI surveys to include all eligible children in the denominator of valid coverage, even though the numerator can only include children who show cards with dates. For example, if 40% of children showed an HBR and 90% of children with HBRs showed evidence of receiving a valid dose of measles, the valid coverage estimate would be 36% (or 90% of 40%). Including all children in the denominator puts the valid coverage estimate on the same scale as crude coverage, but if HBR availability is low, it suppresses the maximum possible value for valid coverage. If only a small portion of children have or show HBRs, then we can only confirm the validity of doses for a small portion of respondents. The White Paper recommends that valid coverage include all eligible children in the denominator but that it only be presented when at least 80% of children have an HBR. |
| Evidence from tick marks | In the best case, doses are documented on HBRs and FBRs with a legible date of vaccination. In some cases, the date is missing and the HBR includes only a check mark or a signature from the vaccinator. In other case the date is present, but incomplete or impossible to read. These children receive credit for a crude dose of the vaccine, but because we cannot know the age at which they received it, they are excluded from indicators that assess age or eligibility at the time of vaccination. |
| Imperfect date values | In some cases, the dates of birth or vaccination may be incorrect on the source document, the survey questionnaire or the survey dataset. One important step during data cleaning is to identify impossible or unlikely dates of birth or vaccination dates and, if paper forms were used, send them back to the data entry team to confirm or correct. The MICS/NICS and PMCCS interviewers entered responses directly into tablets via computer-assisted personal interviewing (CAPI) so there was no possibility of correcting nonsensical dates. In VCQI analyses of RI surveys, imperfect dates are handled thus:   - The analyst provides input parameters listing the earliest and latest possible dates of vaccination for a child in the dataset - Any date of birth that falls outside the appropriate calendar range for the target age group is set to missing (.); those children will not contribute meaningfully to analyses that rely on calculating age at vaccination - Any vaccination date that falls a) before the child’s date of birth, or b) outside the earliest-to-latest window is analysed as if it were a tick mark instead of a date; that is, the child is given credit for crude coverage but is not included in date-based analyses for that dose - Any vaccination dates in a dose series that is out-of-chronological order is also analysed as if it were a tick mark - If the HBR or FBR includes dates for later doses in a series (e.g., OPV2) but is missing evidence for the earlier dose (OPV1) then VCQI analyses the earlier dose as if it had been recorded with a tick mark - Finally, any date that is partially recorded (i.e., includes month and year but missing day) or illegibly recorded, and any date that is legible but nonsensical, like February 30 or September 31 is analysed as if it had been recorded with a tick mark |
| Missing values, 'unsure', and 'do not know' responses | The VCQI Results Quick Interpretation Guide lists how missing/unsure/do not know responses are handled for each indicator [27]. For coverage outcomes, the child is assumed to be unvaccinated if the caretaker is not sure. In cases where the caretaker is confident that the child received the antigen but are unsure how many doses they received, VCQI gives credit for only a single dose, which is a conservative approach. |
| Confidence interval calculations | Symmetric Wald-type confidence intervals can yield absurd lower or upper bounds that fall outside the 0-100% range when the estimated coverage is very low or very high. Several alternative formulae have nice properties:   - Their bounds always fall between 0-100% (inclusive) - If the survey is unbiased, the confidence intervals contain the true population proportion nearly 95% of the time - The intervals are narrow (precise) when compared to alternatives   A coverage survey report should document which formula was used. VCQI can calculate logit, Wilson, Jeffreys, or Clopper-Pearson intervals, and it adjusts the degrees of freedom to account for the complex sample design. The analyses reported here used Wilson confidence intervals when coverage was between 0 and 100% and Clopper-Pearson intervals when coverage was exactly 0% or 100%. |
| How many decimal places to report | It is common to report coverage percentages with a single digit after the decimal place (e.g., 53.4%). But to keep tables from being too wide to fit on a standard page, some reports omit that final digit, rounding to the nearest percent, reasoning that the decimal digit is usually not critical for policymakers. The MICS report and PMCCS reports included the final digit and the NICS report omitted it. |
