# Supplementary material for: Implementing WHO guidance on conducting and analysing vaccination coverage cluster surveys: Two examples from Nigeria
Source: PLoS One. 2021 Feb 26;16(2):e0247415. doi: 10.1371/journal.pone.0247415 (PMC7909665; doi:10.1371/journal.pone.0247415)
Supplement: Attachment — Submitted filename: Response to Reviewers.docx [file pone.0247415.s015.docx]

Sunday November 29^th^, 2020

Olalekan Uthman

Academic Editor,

PLOS ONE

Re: Manuscript ID PONE-D-20-18456

**Title:** Implementing WHO guidance on conducting and analysing vaccination coverage cluster surveys: Two examples from Nigeria

**Authors:** John Ndegwa Wagai, Dale A. Rhoda, Mary L. Prier, Mary Kay Trimner, Caitlin B. Clary, Joseph Oteri, Bassey Okposen, Adeyemi Adeniran, M. Carolina Danovaro-Holliday, Felicity T. Cutts

Dear Olalekan:

It is with enthusiasm that we are sending you our revised manuscript. We have carefully addressed all comments raised by the Academic Editor and the reviewers.

Below you will find in bold-type responses to all comments made by the Academic Editor and reviewers. We have also prepared and submitted a marked-up copy highlighting changes made and an unmarked manuscript where changes have been incorporated.

Hoping that the modifications are to your satisfaction, we look forward to hearing from you.

Best regards,

John Wagai

*Journal Requirements:*

*When submitting your revision, we need you to address these additional requirements.*

*1. Please ensure that your manuscript meets PLOS ONE's style requirements, including those for file naming. The PLOS ONE style templates can be found at*

[*https://journals.plos.org/plosone/s/file?id=wjVg/PLOSOne_formatting_sample_main_body.pdf*](https://journals.plos.org/plosone/s/file?id=wjVg/PLOSOne_formatting_sample_main_body.pdf) *and*

[*https://journals.plos.org/plosone/s/file?id=ba62/PLOSOne_formatting_sample_title_authors_affiliations.pdf*](https://journals.plos.org/plosone/s/file?id=ba62/PLOSOne_formatting_sample_title_authors_affiliations.pdf)

**We have reviewed the PLOS ONE templates and have made appropriate changes to the manuscript based on the style requirements.**

*2. Thank you for stating the following in the Financial Disclosure section:*

*'DAR and MLP and MKT were funded via BMGF Investment ID 29065.  FTC received consultancy fees from BMGF.  CBC is funded on BMGF Investment ID 53009.  MCDH is a WHO staff member. JNW was funded as the primary WHO consultant on the MICS/NICS and on the PMCCS.  
The content is solely the responsibility of the authors and does not necessarily represent the official views of the funding agencies. M. Carolina Danovaro[-Holliday] works for the World Health Organization. The author alone is responsible for the views expressed in this publication and they do not necessarily represent the decisions, policy or views of the World Health Organization. The funders did not play any role in the collection, analysis, interpretation, writing of final reports, or decision to submit this research.*

[*https://www.gatesfoundation.org'*](https://www.gatesfoundation.org')

*We note that one or more of the authors are employed by a commercial company: Independent Consultant, and Biostat Global Consulting.*

*a. Please provide an amended Funding Statement declaring this commercial affiliation, as well as a statement regarding the Role of Funders in your study. If the funding organization did not play a role in the study design, data collection and analysis, decision to publish, or preparation of the manuscript and only provided financial support in the form of authors' salaries and/or research materials, please review your statements relating to the author contributions, and ensure you have specifically and accurately indicated the role(s) that these authors had in your study. You can update author roles in the Author Contributions section of the online submission form.*

*Please also include the following statement within your amended Funding Statement.*

*“The funder provided support in the form of salaries for authors [insert relevant initials], but did not have any additional role in the study design, data collection and analysis, decision to publish, or preparation of the manuscript. The specific roles of these authors are articulated in the ‘author contributions’ section.”*

*If your commercial affiliation did play a role in your study, please state and explain this role within your updated Funding Statement.*

**We agree with this observation. The funding statement has been amended appropriately.**

*b. Please also provide an updated Competing Interests Statement declaring this commercial affiliation along with any other relevant declarations relating to employment, consultancy, patents, products in development, or marketed products, etc.*

*Within your Competing Interests Statement, please confirm that this commercial affiliation does not alter your adherence to all PLOS ONE policies on sharing data and materials by including the following statement: "This does not alter our adherence to PLOS ONE policies on sharing data and materials.” (as detailed online in our guide for authors* [*http://journals.plos.org/plosone/s/competing-interests*](about:blank)*). If this adherence statement is not accurate and there are restrictions on sharing of data and/or materials, please state these. Please note that we cannot proceed with consideration of your article until this information has been declared.*

**We agree with this observation. The Competing Interest Statement has been updated.**

*c. Please include both an updated Funding Statement and Competing Interests Statement in your cover letter. We will change the online submission form on your behalf.*

*Please know it is PLOS ONE policy for corresponding authors to declare, on behalf of all authors, all potential competing interests for the purposes of transparency. PLOS defines a competing interest as anything that interferes with, or could reasonably be perceived as interfering with, the full and objective presentation, peer review, editorial decision-making, or publication of research or non-research articles submitted to one of the journals. Competing interests can be financial or non-financial, professional, or personal. Competing interests can arise in relationship to an organization or another person. Please follow this link to our website for more details on competing interests:* [*http://journals.plos.org/plosone/s/competing-interests*](http://journals.plos.org/plosone/s/competing-interests)

**We have updated the cover letter with an updated Funding Statement and Competing Interests Statement.**

*3. We note that Figures in your submission contain map images which may be copyrighted.*

*All PLOS content is published under the Creative Commons Attribution License (CC BY 4.0), which means that the manuscript, images, and Supporting Information files will be freely available online, and any third party is permitted to access, download, copy, distribute, and use these materials in any way, even commercially, with proper attribution. For these reasons, we cannot publish previously copyrighted maps or satellite images created using proprietary data, such as Google software (Google Maps, Street View, and Earth). For more information, see our copyright guidelines:* [*http://journals.plos.org/plosone/s/licenses-and-copyright*](http://journals.plos.org/plosone/s/licenses-and-copyright)*.*

*We require you to either (a) present written permission from the copyright holder to publish these figures specifically under the CC BY 4.0 license, or (b) remove the figures from your submission:*

*a. You may seek permission from the original copyright holder of Figures to publish the content specifically under the CC BY 4.0 license.*

*We recommend that you contact the original copyright holder with the Content Permission Form (*[*http://journals.plos.org/plosone/s/file?id=7c09/content-permission-form.pdf*](about:blank)*) and the following text:*

*“I request permission for the open-access journal PLOS ONE to publish XXX under the Creative Commons Attribution License (CCAL) CC BY 4.0 (*[*http://creativecommons.org/licenses/by/4.0/*](about:blank)*). Please be aware that this license allows unrestricted use and distribution, even commercially, by third parties. Please reply and provide explicit written permission to publish XXX under a CC BY license and complete the attached form.”*

*Please upload the completed Content Permission Form or other proof of granted permissions as an "Other" file with your submission.*

*In the figure caption of the copyrighted figure, please include the following text: “Reprinted from [ref] under a CC BY license, with permission from [name of publisher], original copyright [original copyright year].”*

*b. If you are unable to obtain permission from the original copyright holder to publish these figures under the CC BY 4.0 license or if the copyright holder’s requirements are incompatible with the CC BY 4.0 license, please either i) remove the figure or ii) supply a replacement figure that complies with the CC BY 4.0 license. Please check copyright information on all replacement figures and update the figure caption with source information. If applicable, please specify in the figure caption text when a figure is similar but not identical to the original image and is therefore for illustrative purposes only.*

*The following resources for replacing copyrighted map figures may be helpful:*

*USGS National Map Viewer (public domain):* [*http://viewer.nationalmap.gov/viewer/*](about:blank)

*The Gateway to Astronaut Photography of Earth (public domain):* [*http://eol.jsc.nasa.gov/sseop/clickmap/*](about:blank)

*Maps at the CIA (public domain):* [*https://www.cia.gov/library/publications/the-world-factbook/index.html*](about:blank) *and* [*https://www.cia.gov/library/publications/cia-maps-publications/index.html*](about:blank)

*NASA Earth Observatory (public domain):* [*http://earthobservatory.nasa.gov/*](about:blank)

*Landsat:* [*http://landsat.visibleearth.nasa.gov/*](http://landsat.visibleearth.nasa.gov/)

*USGS EROS (Earth Resources Observatory and Science (EROS) Center) (public domain):* [*http://eros.usgs.gov/#*](about:blank)

*Natural Earth (public domain):* [*http://www.naturalearthdata.com/*](about:blank)

**Both the manuscript and supplement have been updated to say that the authors independently plotted the maps in this manuscript using Stata Version 16 (StataCorp. 2019. *Stata Statistical Software: Release 16*. College Station, TX: StataCorp LLC.) using shapefiles downloaded from https://energydata.info/dataset/nigeria-administrative-boundaries-2017/resource/2342db86-823e-4bf2-920f-58be34968f36 which are licensed under Creative Commons Attribution 4.0. The shapefiles have not been previously copyrighted to our knowledge. The boundaries and names shown and the designations used on maps in this manuscript do not imply the expression of any opinion whatsoever on the part of the authors or of the World Health Organization concerning the legal status of any country, territory, city or area nor of its authorities, or concerning the delimitation of its frontiers or boundaries.**

**The statement on the map image appearing in the supplement that the map image was from DHS 2013 was from an earlier draft of the manuscript where we had considered using an image from that the Nigeria Demographic and Health Survey (DHS) 2013 but was dropped in favour for maps generated by the authors. This statement has been updated in the supplement.**

*4. Please note that in order to use the direct billing option the corresponding author must be affiliated with the chosen institute. Please either amend your manuscript or remove this option (via Edit Submission).*

**This has been addressed.**

*5. Please remove your figures from within your manuscript file, leaving only the individual TIFF/EPS image files, uploaded separately.  These will be automatically included in the reviewers’ PDF.*

**Agreed, this has been addressed.**

*6. Please include captions for your Supporting Information files at the end of your manuscript, and update any in-text citations to match accordingly. Please see our Supporting Information guidelines for more information:* [*http://journals.plos.org/plosone/s/supporting-information*](about:blank)

**We have included a statement at the end of the manuscript to guide the reader on the supporting information and have updated in-text citations accordingly.**

*Reviewers' comments:

Reviewer's Responses to Questions*

***Comments to the Author*** *1. Is the manuscript technically sound, and do the data support the conclusions?

The manuscript must describe a technically sound piece of scientific research with data that supports the conclusions. Experiments must have been conducted rigorously, with appropriate controls, replication, and sample sizes. The conclusions must be drawn appropriately based on the data presented.*

*Reviewer #1: Yes*

*2. Has the statistical analysis been performed appropriately and rigorously?*

*Reviewer #1: Yes*

*3. Have the authors made all data underlying the findings in their manuscript fully available?

The* [*PLOS Data policy*](http://www.plosone.org/static/policies.action#sharing) *requires authors to make all data underlying the findings described in their manuscript fully available without restriction, with rare exception (please refer to the Data Availability Statement in the manuscript PDF file). The data should be provided as part of the manuscript or its supporting information, or deposited to a public repository. For example, in addition to summary statistics, the data points behind means, medians and variance measures should be available. If there are restrictions on publicly sharing data—e.g. participant privacy or use of data from a third party—those must be specified.*

*Reviewer #1: Yes*

*4. Is the manuscript presented in an intelligible fashion and written in standard English?

PLOS ONE does not copyedit accepted manuscripts, so the language in submitted articles must be clear, correct, and unambiguous. Any typographical or grammatical errors should be corrected at revision, so please note any specific errors here.*

*Reviewer #1: Yes*

*5. Review Comments to the Author

Please use the space provided to explain your answers to the questions above. You may also include additional comments for the author, including concerns about dual publication, research ethics, or publication ethics. (Please upload your review as an attachment if it exceeds 20,000 characters)*

*Reviewer #1: Dear Authors,

This is an interesting topic and properly researched work, however, there are some concerns. Please find below my comments:


Abstract
1. The abstract did not highlight the findings of the surveys, the differences and importance of using either of the two.
2. What is the purpose of this study?
3. Highlight priority areas for action.*

**This has been acted upon. We have revised the abstract and outlined the purpose of the study and priority areas for action.**

*Background
1. Line 40: Meaning of DTP?
2. Be consistent with the use of GAVI or Gavi.
3. Line 51: The references should be: (9-10)
4. Brief description of VCQI is needed.
5. What are the specific challenges of implementing a high-quality household survey?
6. Line 60: Please identify the key partners.*

**In the manuscripts background we have addressed the issues raised including adding the meaning of DPT (containing diphtheria-tetanus-pertussis), used the word Gavi consistently throughout the manuscript and corrected the references.**

**We have briefly described the Vaccination Coverage Quality Indicators (VCQI) software that is used in the analysis of vaccination coverage data and provided citation for additional documentation on VCQI.**

**Specific challenges in the implementation of high-quality household surveys have been listed. In addition, citation to literature on the challenges of implementing vaccination coverage household surveys are provided.**

**Key partners involved in drafting a white paper on the harmonization of collection and analysis of vaccination coverage data by survey programs have been listed.**

*Methods
1. Ethical considerations of the initial data collection process for the two surveys were not mentioned.
2. Line 117: Please write the full meaning of SIA.*

**Supplemental Immunisation Activity (SIA) has been described in full.**

**We have provided a description of the ethical considerations taken while conducting the initial data collection process.**

*Results
1. There is need to define and differentiate state data from regional data when reporting. Sokoto and Lagos are states while North West or North East are regions. It is confusing for someone who is not conversant with Nigeria’s geopolitical structure to understand the differences without proper definition of these variables.*

**In VCQI, results tables and figures are presented in strata that can be customised by the user. In the Nigeria example, we show national, zonal and state results in the same figure so that results for each state can easily be compared to the average in its zone and the national average. National and zonal names have been capitalised and horizonal lines have been used to demarcate different zones. These style and formatting options can be changed by the user according to their needs. Text has been added to the manuscript to guide readers who may not be familiar with the Nigerian geopolitical context on how to read the tables and figures.**

*2. Lines 244 – 252: It will be nice having tables to showcase this statements and possibly univariate regression to show if there are any significant differences.*

**We have pointed the reader to the MICS/NICS report with original tabulations.**

*Discussion
1. Line 470: MOV varies across the country, what are the likely reasons for these differences.
2. Recall bias is also a major limitation.
3. Line 488: Please re-phrase the sentence.*

**We have discussed the likely causes of MOV and suggested that the causes of MOVs can be objectively investigated by use of properly conducted health facility surveys as recommended by the World Health’s Organisation toolkit on MOVs.** <https://www.who.int/immunization/programmes_systems/policies_strategies/MOV/en/>

**The statement that originally appeared in line 488 has been rewritten.**

*Figures and tables
1. Most of the figure and table titles are rather too long.
2. Figures 1, 4, 6 and 7: The states and regions should be clearly defined and separated. The state, regional and national data were mixed up.*

**While we were striving to achieve clarity in the titles for our tables and figures, we have reduced the length of the longest titles. In addition, we have added a description why state level results have been presented next to the zone level and national averages for ease of comparison.**

*6. PLOS authors have the option to publish the peer review history of their article (*[*what does this mean?*](https://journals.plos.org/plosone/s/editorial-and-peer-review-process#loc-peer-review-history)*). If published, this will include your full peer review and any attached files.


If you choose “no”, your identity will remain anonymous but your review may still be made public.*


***Do you want your identity to be public for this peer review?*** *For information about this choice, including consent withdrawal, please see our* [*Privacy Policy*](https://www.plos.org/privacy-policy)*.*

*Reviewer #1: No*

*[NOTE: If reviewer comments were submitted as an attachment file, they will be attached to this email and accessible via the submission site. Please log into your account, locate the manuscript record, and check for the action link "View Attachments". If this link does not appear, there are no attachment files.]

While revising your submission, please upload your figure files to the Preflight Analysis and Conversion Engine (PACE) digital diagnostic tool,* [*https://pacev2.apexcovantage.com/*](https://pacev2.apexcovantage.com/)*. PACE helps ensure that figures meet PLOS requirements. To use PACE, you must first register as a user. Registration is free. Then, login and navigate to the UPLOAD tab, where you will find detailed instructions on how to use the tool. If you encounter any issues or have any questions when using PACE, please email PLOS at* [*figures@plos.org*](mailto:figures@plos.org)*. Please note that Supporting Information files do not need this step.*

**Files have been uploaded into the PACE engine and corrected versions will be submitted.**
